# A retrospective analysis of real-world use of the eaTracker® My Goals website by adults from Ontario and Alberta, Canada

**DOI:** 10.1186/s12889-016-3640-6

**Published:** 2016-09-15

**Authors:** Jessica R. L. Lieffers, Helen Haresign, Christine Mehling, Rhona M. Hanning

**Affiliations:** 1 School of Public Health and Health Systems, University of Waterloo, 200 University Avenue West, Waterloo, Ontario N2L 3G1 Canada; 2 EatRight Ontario/Dietitians of Canada, 480 University Avenue, Suite 604, Toronto, Ontario M5G 1V2 Canada

**Keywords:** Goals, Website, Diet, Exercise, Program Evaluation, Adult, Canada

## Abstract

**Background:**

Little is known about use of goal setting and tracking tools within online programs to support nutrition and physical activity behaviour change. In 2011, Dietitians of Canada added “My Goals,” a nutrition and physical activity behaviour goal setting and tracking tool to their free publicly available self-monitoring website (eaTracker® (http://www.eaTracker.ca/)). My Goals allows users to: a) set “ready-made” SMART (Specific, Measurable, Attainable, Realistic, Time-related) goals (choice of *n =* 87 goals from *n =* 13 categories) or “write your own” goals, and b) track progress using the “My Goals Tracker.” The purpose of this study was to characterize: a) My Goals user demographics, b) types of goals set, and c) My Goals Tracker use.

**Methods:**

Anonymous data on all goals set using the My Goals feature from December 6/2012-April 28/2014 by users ≥19y from Ontario and Alberta, Canada were obtained. This dataset contained: anonymous self-reported user demographic data, user set goals, and My Goals Tracker use data. Write your own goals were categorized by topic and specificity. Data were summarized using descriptive statistics. Multivariate binary logistic regression was used to determine associations between user demographics and a) goal topic areas and b) My Goals Tracker use.

**Results:**

Overall, *n =* 16,511 goal statements (75.4 % ready-made; 24.6 % write your own) set by *n =* 8,067 adult users 19-85y (83.3 % female; mean age 41.1 ± 15.0y, mean BMI 28.8 ± 7.6kg/m^2^) were included for analysis. Overall, 33.1 % of ready-made goals were from the “Managing your Weight” category. Of write your own goal entries, 42.3 % were solely distal goals (most related to weight management); 38.6 % addressed nutrition behaviour change (16.6 % had unspecific general eating goals); 18.1 % addressed physical activity behaviour change (47.3 % had goals without information on exercise amount and type). Many write your own goals were poor quality (e.g., non-specific (e.g., missing amounts)), and possibly unrealistic (e.g., no sugar). Few goals were tracked (<10 %). Demographic variables had statistically significant relations with goal topic areas and My Goals Tracker use.

**Conclusions:**

eaTracker® users had high interest in goal setting and the My Goals feature, however, self-written goals were often poor quality and goal tracking was rare. Further research is needed to better support users.

**Electronic supplementary material:**

The online version of this article (doi:10.1186/s12889-016-3640-6) contains supplementary material, which is available to authorized users.

## Background

Many Canadian adults do not meet nutrition [1] and physical activity [2] recommendations associated with good health. Further, the 2012 to 2013 Canadian Health Measures Survey found that 28 % of women, and 43 % of men 18–79 years of age were overweight (body mass index (BMI) ≥ 25kg/m^2^) and 26 % of women, and 27 % of men in the same age range were obese (BMI ≥ 30kg/m^2^) [3]. Previous work has also found that poor nutrition and physical inactivity are responsible for substantial fractions of chronic diseases (e.g., diabetes, heart disease) [4–7]. In fact, in 2012, five of the top ten causes of death in Canadians were chronic non-communicable diseases with known linkages to poor nutrition and/or physical activity behaviours and excess body weight (i.e., heart disease, cancer, diabetes, stroke, kidney disease) [8]. The high prevalence of individuals failing to meet nutrition, physical activity, and body weight recommendations associated with optimal health are major public health concerns. Ways to improve these types of behaviours are important for the public health agenda in Canada and many other jurisdictions.

Numerous approaches are available to help individuals improve their nutrition and/or physical activity behaviours [9]. Techniques surrounding goals, which are targets where individuals can direct efforts, including prompt goal setting and prompt review of goals are frequently used for this purpose [10]. Further, goal setting is a key component of behavioural therapy for weight [11, 12], and diabetes [13] management, 5As for obesity treatment in primary care [14, 15], and many other behaviour change approaches described elsewhere [16]. Much of what we know about how goals influence behaviours comes from Goal Setting Theory developed by Locke, Latham and colleagues [17, 18] which was adapted for healthcare by Stretcher et al [19]. This theory suggests that specific and difficult (but possible) goals support better outcomes than vague “do your best” goals; that goals impact performance by directing efforts, being energizing, enhancing persistence, and promoting use and discovery of goal specific skills and knowledge; and that behaviours are moderated by goal commitment, goal importance, self-efficacy, feedback and task complexity [17, 18]. The SMART acronym (“Specific,” “Measurable,” “Attainable,” “Realistic,” and “Time-related”) [20] is frequently used to bridge theory and practice [21–23].

Several review articles have examined interventions using goals for nutrition and/or physical activity behaviour change in an attempt to understand how goals are used (e.g., types of goals used, how goals were chosen, inclusion of rewards and/or feedback) and the effectiveness of such interventions in different areas including primary care [16], weight management [24], and nutrition and physical activity behaviour change in general [25, 26]. Generally, these reviews find that studies incorporating goal setting can be effective; however, knowledge of specific aspects of the goal setting process that are associated with the best outcomes are not well understood. Nevertheless, goal setting does appear to be worthy of use in these types of interventions.

Although goals are a familiar concept to many individuals because of use in different settings (e.g., workplace, sports), previous studies have identified challenges when using them in health situations in the absence of or with limited health professional support (e.g., individuals may set poor quality goals or long term distal goals only (e.g., lose 100lbs, live a long life) that are broad and non-specific, with no timeframe or achievement plan)) [27, 28]. In-person health professional assistance with goals is perhaps the most traditional way to provide support with this technique, however, some individuals may not have easy access to this support (e.g., cost, located in remote areas, long wait times). Moreover, clinicians have been found to be hesitant to provide this service to their patients which has been summarized elsewhere [16]. Electronic tools (e.g., websites) have gained momentum for nutrition and/or physical activity behaviour change and weight management interventions [29, 30] and have the potential to assist users with goal setting and tracking both in the presence and absence of professional support. These tools can provide support with goal use in numerous ways ranging from goal setting education (e.g., as part of modules, tutorials) to online goal setting and tracking tools [31–38]. Moreover, website-based interventions incorporating goal-based tools have been associated with positive outcomes when tested in research trial settings [34, 39]. However, usage of such tools outside of supportive research trial settings is largely unknown.

eaTracker® (http://www.eatracker.ca/) (Dietitians of Canada, Toronto, Canada) is a free, publicly available Canadian nutrition and physical activity behaviour self-monitoring website. Members of the general public may find out about eaTracker® through various channels including via their dietitian or other health professional (as the tool is well known amongst Canadian health professionals interested in nutrition), health and professional organizations (e.g., Dietitians of Canada), Internet searches, government, and school courses. “My Goals” was added to eaTracker® in 2011 that allows users to set: a) “ready-made” SMART goals to be completed weekly (choice of *n =* 87 goals within *n =* 13 categories [Additional file 1]; e.g., “Avoid all fried foods this week”) and b) “write your own” goals which includes the requirement that users choose a frequency (daily, weekly, monthly, once by an end-date) from a drop-down menu. The “My Goals Tracker” allows users to self-identify goal progress as “Met My Goal” or “Still Trying” (tracking is available by beginning of the next day, week, month, or end-date for daily, weekly, monthly, and one-time goals, respectively). “My Goals” also contains goal progress logs (“Manage My Goals” and “My Success”). Ontario, Canada users were also provided information on writing SMART goals. My Goals screen shots are shown in Additional file 2. In December 2012, EatRight Ontario, a dietitian advisory service that provides residents of Ontario, Canada with free access to healthy eating advice from registered dietitians by phone, email and website (http://www.eatrightontario.ca/), added optional free supports for Ontario My Goals users including: a) emailed and eaTracker® delivered motivational messages (~1/week) with tips, recipes, web-links usually specific to ready-made goals or general messages for write your own goals (e.g., reminders to log into eaTracker®) [screenshots shown in Additional file 2], and b) assistance with goals by phone or e-mail from contact centre dietitians.

Data from users of the My Goals feature, including user demographics and the types of goals set, provided an ideal opportunity to study natural use of a publicly available website that allows users to set and track both website-provided and their own written goals. This information is relevant to nutrition, public health and information technology professionals interested in incorporating goal setting and tracking tools as part of website-based interventions for nutrition and physical activity behaviour change to help decrease the burden of chronic disease. Further, this information provides insight into the needs of public users of these types of tools. Using anonymous retrospective data collected from users of the My Goals feature, the purpose of this study was to characterize a) My Goals feature users, b) goal type, topic, specificity, and c) My Goals Tracker use.

## Methods

The University of Waterloo Office of Research Ethics provided ethics approval. This was a secondary analysis of anonymous data on all goals set from December 6, 2012-April 28, 2014 by active eaTracker® My Goals users as of April 28, 2014, ≥19 years of age, from Alberta and Ontario, Canada. Anonymous user self-reported demographics (age, sex, height, weight, pregnancy status, breastfeeding status, physical activity level, province of residence) when goals were set were also acquired. Goals both set and deleted on the same day were excluded from all analyses. The eaTracker® website privacy policy specifies to users that anonymous data may be provided to researchers to create reports and collect statistics which was the case for this project; additional written consent was not obtained for this anonymous retrospective data analysis.

Body mass index (BMI) was calculated using self-reported height and weight. Users with implausible height (<1.22m or >2.13m) and/or weight (<34kg or >227kg) values [40] were excluded from BMI analyses only. For all analyses in this paper, user demographics were captured when individuals set their first included goal with the My Goals feature within the data capture window (December 6, 2012-April 28, 2014).

### Write your own goal analysis

Related studies characterizing health-related goal content [41, 42] helped guide this analysis. Nutrition behaviour change goals (e.g., drink more milk) were categorized based on topic and specificity (usually quantity). When quantity did not fit with goal topic (e.g., portion size, self-monitoring), a goal was considered specific when additional details were provided other than only behaviour identification (e.g., how or when the behaviour would be conducted e.g., “using portion controlled foods” vs. “portion control”). Goals to follow a specific diet (e.g., eat a vegetarian diet) were only categorized by topic area, not specificity. Physical activity goals were categorized by both activity type and amount; activity self-monitoring (e.g., track activity) was categorized as other-specific. Goals to use eaTracker® were categorized under both nutrition and activity self-monitoring. Long term (distal) outcome goals (e.g., lose weight, manage diabetes, gain muscle) were categorized by topic area only.

Each goal was categorized once using the most relevant category (with the exception of goals to use eaTracker® as described above), and write your own goal statements with >1 goal (e.g., a single statement containing goals to drink milk, eat more vegetables, and lose weight) were categorized as separate goals with a maximum of one occurrence of a specific category for each write your own goal statement. Time was not considered in this analysis because a frequency was chosen by the user for all write your own goals.

A codebook was created and used to categorize all write your own goals; JL created the codebook and categorized all goals. Topic area categories were generated inductively from the data; however, Canada’s Food Guide [43] was used to guide organization of goals relevant to this document. Goals that were identified as difficult by JL were discussed with RH and consensus was achieved. In addition, two second researchers (dietitian PhD students) reviewed the codebook and re-categorized a subset of goals (10 %); any discrepancies were discussed and consensus was achieved.

### Statistical analysis

All statistics were completed using SPSS versions 22 and 23 (IBM Corp., Armonk, New York). All continuous variables are presented as mean ± standard deviation. All p values were two tailed and p values were considered significant if <0.05. Chi-square tests (categorical variables), and independent sample t-tests (continuous variables) were used to compare user demographics.

Multivariate binary logistic regression was used to determine the relationship between the types of goals set and demographic variables. Goal type was the dependent variable (i.e., set specific goal type/did not set specific goal type) and sex, age, BMI, pregnancy status, breastfeeding status, and self-reported activity were independent variables. Ready-made and write your own goals were sometimes grouped together for this analysis when topic areas overlapped (e.g., vegetables and fruit, planning and preparing food); however, some write your own goals were kept on their own if their topic area was not covered as part of the ready-made goals. Write your own long term (distal) outcome goals (e.g., weight loss, body composition change) were not grouped with any ready-made goals. Categories with <30 goals set in total (ready-made and write your own goals combined) as well as those which group several different types of goals together (e.g., other) were not included in this analysis. Multivariate binary logistic regression was also used to determine the relationship between My Goals Tracker use (i.e., used tracker ≥1 time vs. never used tracker) and demographic variables.

## Results

In total, *n =* 16,511 goal statements (75.4 % (12,449/16,511) were ready-made; 24.6 % (4,062/16,511) were write your own) set by *n =* 8,067 users 19-85 years of age were included for analysis. A cut off of 85 years of age was chosen as only *n =* 6 goal statements were written by users >85 years of age with the next youngest user being 96 years of age. Of note, there were ~ *n =* 29,685 eaTracker® accounts active between December 6, 2012 and April 28, 2014 belonging to users 19-85 years of age from Ontario and Alberta who last logged during and after that date range (this number was obtained on October 2, 2015). In total, *n =* 16,375 and *n =* 136 goal statements were in English and French, respectively.

Demographics when users set their first goal are presented in Table 1; overall, the mean user BMI was over 25kg/m^2^, mean age was 41.1 ± 15.0 years and over 80 % of users were female. Ontario users who had signed up for EatRight Ontario messaging were more often female, less active, and on average were older and had a higher BMI versus Alberta users and Ontario users who had not signed up for EatRight Ontario messaging (results not shown).

**Table 1:** User demographics

	All users (*n =* 8,067)	Female (*n =* 6,717)	Male (*n =* 1,350)	*p* value^b^
Province of Residence				
Ontario – non EatRight Ontario message user	4,499 (55.8 %)	3,628 (54.0 %)	871 (64.5 %)	<0.001^c^
Ontario – EatRight Ontario message user	2,195 (27.2 %)	1,938 (28.9 %)	257 (19.0 %)	
Alberta user	1,373 (17.0 %)	1,151 (17.1 %)	222 (16.4 %)	
Age (years)	41.1 ± 15.0	41.4 ± 14.7	39.8 ± 16.5	0.001
Age Category (years)				
19–30	2,521 (31.3 %)	1,987 (29.6 %)	534 (39.6 %)	<0.001
31–50	2,981 (37.0 %)	2,580 (38.4 %)	401 (29.7 %)	
51–70	2,436 (30.2 %)	2,058 (30.6 %)	378 (28.0 %)	
71–85	129 (1.6 %)	92 (1.4 %)	37 (2.7 %)	
BMI (kg/m^2^)^a^	28.8 ± 7.6	28.8 ± 7.7	28.7 ± 6.8	0.7
BMI Category (n ( %))^a^				
<18.5	162 (2.0 %)	140 (2.1 %)	22 (1.6 %)	<0.001
18.5–24.9	2,684 (33.5 %)	2,295 (34.4 %)	389 (29.0 %)	
25.0–29.9	2,291 (28.6 %)	1,823 (27.3 %)	468 (34.9 %)	
≥30	2,872 (35.9 %)	2,409 (36.1 %)	463 (34.5 %)	
Activity Level (n ( %))				
Sedentary	1,242 (15.4 %)	1,071 (15.9 %)	171 (12.7 %)	<0.001
Low Active	4,106 (50.9 %)	3,549 (52.8 %)	557 (41.3 %)	
Active	2,345 (29.1 %)	1,840 (27.4 %)	505 (37.4 %)	
Very Active	344 (4.3 %)	229 (3.4 %)	115 (8.5 %)	
Not Specified	30 (0.4 %)	28 (0.4 %)	2 (0.1 %)	
Pregnant (n (%))				
No		6,594 (98.2 %)		
Yes		123 (1.8 %)		
Breastfeeding (n (%))				
No		6,585 (98.0 %)		
Yes		132 (2.0 %)		
Types of Goals Set (n ( %))				
Ready-made only	5,480 (67.9 %)	4,540 (67.6 %)	940 (69.6 %)	0.001
Write your own only	1,507 (18.7 %)	1,236 (18.4 %)	271 (20.1 %)	
Both	1,080 (13.4 %)	941 (14.0 %)	139 (10.3 %)	
Average Number of Goals Set	2.0 ± 1.3 Median: 2 Range: 1–20	2.1 ± 1.3 Median: 2 Range: 1–20	1.9 ± 1.2 Median: 1 Range: 1–15	<0.001
Ready-made goals	1.5 ± 1.3	1.6 ± 1.3	1.4 ± 1.2	<0.001
Write your own goals	0.5 ± 1.0	0.5 ± 1.0	0.4 ± 0.9	0.01

Province of Residence, age, sex, BMI, activity level, pregnancy status, and breastfeeding status were self-reported

^a^BMI data were based on self-reported height and weight and available for *n =* 8,009 (*n =* 6,667 females; *n =* 1,342 males) users

^b^chi-square test or independent samples *t*-test to test differences between males and females

^c^chi-square ﻿test only included Ontario users

On average, users had ~2 goal statements; males set less goals compared to females. Among users who had ≥2 goal statements (*n =* 4,485), 91.1 % set all goals on the same day. Only *n =* 547 (*n =* 323 ready-made; *n =* 224 write your own) goal statements were ever deleted.

### Ready-made goals

Descriptive statistics of ready-made goals organized by category are presented in Table 2. In total, 81.3 % (6,560/8,067) of users had set ≥1 ready-made goal(s). Overall, these *n =* 6,560 users set on average 1.9 ± 1.2 ready-made goals (range: 1–20) and 56.4 % (3,697/6,560) chose ≥1 goal from the “Managing your Weight” category; this category encompassed 33.1 % (4,116/12,449) of all ready-made goals set. The most popular goals were: “Avoid snacking while reading, using the computer or watching TV every day this week” (9.4 % (1,170/12,449) of all ready-made goals set), “Make a menu plan and shopping list and use it this week” (6.5 % (805/12,449)), and “Avoid second helpings during meals and snacks every day this week” (5.9 % (734/12,449)).

**Table 2:** Types of ready-made goals set and tracked by My Goals feature users organized using the different ready-made goal categories

	Total number of goals set from the specified goal category ( % of all ready-made goals set)^a^	Total number of users who had set a goal from the specified goal category ( % all users who set ready-made goals)^b^	Total number of goals tracked from the specified goal category ( % of all goals tracked from the specified goal category)^c^
Goal Category			
Managing your weight	4,116 (33.1 %)	3,697 (56.4 %)	277 (6.8 %)
Getting active	1,634 (13.1 %)	1,527 (23.3 %)	131 (8.1 %)
Choosing more vegetables and fruit	1,622 (13.0 %)	1,555 (23.7 %)	104 (6.5 %)
Eating less fat, sugar, and sodium	1,380 (11.1 %)	1,306 (19.9 %)	86 (6.3 %)
Planning and preparing food	1,291 (10.4 %)	1,235 (18.8 %)	72 (5.7 %)
Eating a healthy breakfast	643 (5.2 %)	629 (9.6 %)	60 (9.4 %)
Getting more fibre	411 (3.3 %)	390 (5.9 %)	28 (6.9 %)
Eating a healthy dinner	295 (2.4 %)	287 (4.4 %)	17 (5.8 %)
Eating a healthy lunch	284 (2.3 %)	280 (4.3 %)	23 (8.2 %)
Choosing healthier beverages	254 (2.0 %)	247 (3.8 %)	22 (8.9 %)
Choosing healthier grain products	223 (1.8 %)	220 (3.4 %)	14 (6.4 %)
Choosing healthier meat and alternatives	215 (1.7 %)	199 (3.0 %)	13 (6.2 %)
Choosing healthier milk and alternatives	81 (0.7 %)	77 (1.2 %)	8 (10.3 %)

^a^ *n =* 12,449 ready-made goals were set in total; *n =* 12,449 was the denominator used to calculate all percentages in this column

^b^ *n =* 6,560 users in total had set ready-made goals; *n =* 6,560 was the denominator used to calculate all percentages in this column

^c^Because the My Goals Tracker was only available to track goals at the start of the next week for ready-made goals, only goals that were active for ≥7 days were included as part of the denominator for the percentage of all goals tracked from the specified goal category

Few ready-made goals were tracked with the My Goals Tracker. For ready-made goals that were active for ≥7 days (*n =* 12,268) (note: active for ≥7 days means that goals were not deleted within the first seven days of being set, and were set at least seven days prior to data request date (i.e., April 28, 2014)), only 7.0 % (855/12,268) had been tracked ≥1 time(s) (note: only 20.9 % (179/855) of those goals were tracked more than once).

### Write your own goals

Overall, 32.1 % (2,587/8,067) of users set ≥1 write your own goal statement(s) which usually encompassed distal, nutrition, and/or physical activity behaviour change goals. Further, some write your own goal statements encompassed related health behaviour change goals (e.g., sleep, smoking, anger, stress, taking time for one’s self, meditation, attitude, medication) (*n =* 56), body measurement (usually weight), blood glucose, or medication self-monitoring (*n =* 16), and general healthy living (sometimes to lose weight or be healthier) (*n =* 19). In addition, *n =* 553 statements contained goals from more than one category (e.g., eat vegetables, lose weight). Of note, *n =* 95 full write your own goal statements were irrelevant to health or nonsense. Table 3 provides example write your own behaviour change goals organized by topic and specificity (if applicable).

**Table 3:** Examples of write your own physical activity and nutrition behaviour change goals by topic and specificity

	Specific (e.g., with amount)	Less specific (e.g., with amount)
*Physical Activity Behaviour Change Goals*		
Aerobic (e.g., running, swimming etc.)	Get 10,000 steps a day, Run 30 min 6 times this week	Moderate to vigorous activity 3 times per week, Run daily
Strength and flexibility (e.g., weights, yoga, Pilates etc.)	Do 15 min of activity 1 time this week in the Weight room (weights, resistance training), Go to one yoga class per week,	I would like to start weight training 2-3 times a week
Unknown exercise	Workout 30 min a day, Exercise 150min per week	Exercise more, Be more active, Exercise daily
*Nutrition Behaviour Change Goals*		
Eating Well With Canada’s Food Guide Food Group Goals		
↑/specific serving number amounts	Consume four servings of vegetables daily	Eat more fruit and vegetables
Limit food group foods	No dairy	Eat less bread
Improving quality of food group foods	Two servings fish each week	To eat more beans and legumes instead of meat this month
Limiting unhealthy foods/beverages and/or fat, cholesterol, sodium, and/or sugar in general	No sugar, Avoid junk food, Limit sodium to <1500 mg/d, Give up diet Pepsi	Eat less sodium and fat and sugar, Cut down on eating sweets
Alcohol (limit or specific amount)	Cut alcohol use to a glass of wine/day, Cut out beer	Reduce alcohol intake
Water (increase or specific amount)	Drink 8 cups of water per day	Drink more water
Limit coffee and/or caffeine	Limit my coffee intake to 2 cups/day	Drink less coffee
Eat more/specific amounts of certain nutrients (e.g., protein, fibre, calcium, vitamins D, C, E, iron, potassium, unsaturated fats)	Consume sufficient iron daily, Drink or eat 1000 mg calcium each day	Increase my fibre intake, Increase protein intake at lunch time
Eat less/limit certain nutrients (e.g., carbohydrates) (except fat, sodium, sugar)	Eat only 1/2 cup of starch at supper	Eat less carbs
Caloric/food intake amounts	Consume <1500kcals, No over-eating and no eating my kids left overs!	Eat more calories, Eat less, Limit extra desserts/snacks
Portion control	Weigh and measure my portions, To eat portion controlled foods	Eat smaller portions
Self-monitor diet and/or ↑ awareness	Keep a food diary daily	Be aware of what I am eating.
Eating out less/Eat at home more	I want to only dine out for 1 meal a week	Eat more at home
Evening/night time eating (e.g., healthier and/or restricting)	No eating after 8:00pm	Limit snacks at night
Follow Canada’s Food Guide	Follow Canada’s food guide	
Follow ﻿vegetarian/vegan diets	Eat mostly vegan	
Follow fad diets (not gluten free)	More alkaline and less acidic foods	
Gluten free (includes wheat free)	Follow Celiac diet	
Follow other plans (includes FODMAP guidelines, heart healthy, diabetes, Optifast®, Weight Watchers®, DASH, dietitian meal plans, weight lifting, pregnancy diet, unspecified diet plans)	Following the dietitian’s plan, Eat only my shakes this week, Follow diet, Follow Diabetes diet	
Glycemic index	Keep a low glycemic index diet	
Clean eating (includes unprocessed, whole, fresh, raw, natural foods)	Eating cleaner, Eat as much raw as possible, To eat fresh food	
Supplements	Take 600-800 IU vitamin D daily	
Planning and preparing food^†^	Cook dinner from scratch a minimum of 3 times per week, Plan 4–5 smaller meals per day	Better planning and preparing of food, Plan healthy meals, Plan Meals
Goals describing when food is eaten or not eaten/eating patterns	Eat Breakfast, Lunch, Dinner & Snack every day, Not to eat in between meals	Eat more regularly, Try not to snack so much throughout the day
Breakfast	Eating healthy breakfast	Incorporate cereal or smoothies more often for breakfast
Psychological aspects of eating	Don’t binge, Drink water or 0 cal drink when hungry, Stop emotional eating	To control food intake, Respect food as a means of nutrition and to satisfy hunger
General eating improvements	Eat healthy meals and snacks, Eat the recommended DV of minerals and vitamins, Eat a variety of foods	Eat healthy, eat a balanced diet, eat right to avoid IBS symptoms

Overall, there were *n =* 1,986, *n =* 614, *n =* 233, and *n =* 1,134 daily, weekly, monthly, and one-time write your own goal statements, respectively (excluding irrelevant to health/nonsense goals); these goals were rarely tracked. For daily write your own goal statements active for ≥7 days (*n =* 1,944), 13.4 % (261/1,944) were tracked (39.1 % (102/261) of those were tracked more than once). For weekly write your own goal statements active for ≥7 days (*n =* 605), 13.2 % (80/605) were tracked (41.3 % (33/80) of those were tracked more than once). For monthly write your own goal statements that were active for ≥30 days (*n =* 222), 6.3 % (14/222) were tracked (35.7 % (5/14) of those were tracked more than once). For one-time write your own goal statements active seven days after the end-date (*n =* 911), 4.3 % (39/911) were tracked once.

#### Distal goals

Overall, 42.3 % (1,720/4,062) write your own goal statements were solely distal goals (e.g., weight loss) and *n =* 639 users set only this goal type. An additional *n =* 173 goal statements encompassed both distal and other goal types without forming a direct link (e.g., lose weight and eat healthier). This section will discuss distal goals included in these *n =* 1,893 statements set by *n =* 1,621 users.

Several write your own goal statements (*n =* 1,542) had weight management goals with *n =* 1,382 addressing weight loss (*n =* 1,042 of those specified a total weight loss amount (e.g., number of lb/kg, clothes size, BMI level) or rate). Weight gain goals were less common (*n =* 49 statements had these goals), with *n =* 25 set by males ≤30 years of age.

Weight management (excluding weight gain) goals were rarely linked to a direct reason (e.g., lose weight to manage diabetes). When a direct reason was listed, reasons included to manage health conditions (e.g., diabetes, hypertension, arthritis, back pain, surgery qualification, medication discontinuation) (*n =* 15 statements had these goals), be healthier, feel better, and/or increase energy (*n =* 5), sports (*n =* 3), conception, pregnancy, and/or post-partum (*n =* 3), quitting smoking (*n =* 2), and improving fitness (*n =* 1). Weight management for special events (e.g., vacation, wedding, anniversary, holiday, birthday, and graduation) and fitting better into clothes were also mentioned. When weekly weight loss amounts were directly specified, most were within a prudent 0.5-1kg/week, however, higher amounts were sometimes specified (e.g., lose 1.4kg/week). In addition, there were some users with BMIs in the low normal range who desired weight loss.

In total, *n =* 186 write your own goal statements had body composition improvement goals (e.g., decrease body fat, decrease waist circumference, gain muscle, increase leanness, improve physique, wear certain clothes (e.g., bathing suit) well, be more “toned,” “ripped,” or “bulky,” stronger bones, happy with body). These goals were frequently set by users ≤30 years of age (*n =* 94).

Health issue/disease prevention and/or management goals (excluding *n =* 15 goals directly linked to weight management) were included as part of *n =* 118 statements, *n =* 88 which were formed by users ≥50 years of age. Diabetes or blood glucose control was the most common (*n =* 59), followed by blood lipid or heart disease (*n =* 26), and hypertension (*n =* 13) management; however, other conditions were also mentioned (e.g., pain, anemia, getting through surgery, irritable bowel syndrome, fatty liver, fluid retention, decreasing potassium, bone health, eating disorders).

Fitness improvement goals (e.g., more fit, flexible, stronger, stamina, run 5km, run a marathon, improve posture) were present in *n =* 112 statements. Lastly, *n =* 79 statements had goals to be healthier, increase energy, and feel better, improve self-esteem in general, and *n =* 4 statements had goals to conceive and/or have a healthy pregnancy.

#### Physical activity behaviour change goals

Overall, *n =* 550 write your own goal statements were solely physical activity behaviour change goals; an additional *n =* 184 statements encompassed both physical activity and other goal types without forming a direct link. This section will discuss physical activity behaviour change goals included in these *n =* 734 statements set by *n =* 642 users.

Table 4 classifies physical activity behaviour improvement goals by activity type and specificity. Overall, 47.3 % (347/734) of these statements had a goal to increase activity without specifying amount (e.g., minutes, repetitions) and type; a further 12.1 % (89/734) of statements specified activity amount but not type. Statements with goals to increase aerobic activities (e.g., walking, running, swimming, dancing, biking, elliptical, stairs, jumping jacks, boxing) (*n =* 149 specific; *n =* 85 less specific) outnumbered those to increase strength and flexibility activities (e.g., weights, yoga, Pilates, stretching, core) (*n =* 24 specific; *n =* 52 less specific).

**Table 4:** Types of write your own physical activity and nutrition behaviour change goals set by users of the eaTracker® My Goals feature organized by topic and specificity

		Specific (e.g., with amount) (*n =* number of goal statements with specified goal type)	Less specific (e.g., with amount) (*n =* number of goal statements with specified goal type)
*Physical activity behaviour change goals (n = 734 statements included these goals)*			
Aerobic (e.g., running, swimming etc.)		*n =* 149	*n =* 85
Strength and flexibility (e.g., weights, yoga, Pilates etc.)		*n =* 24	*n =* 52
Unknown exercise		*n =* 89	*n =* 347
Other (e.g., fitness classes, exergaming, activity tracking)		*n =* 27	*n =* 4
*Nutrition behaviour change goals (n = 1,568 statements included these goals)*			
Canada’s Food Guide Food Group Goals			
Vegetables and Fruit	↑/specific serving number amounts	*n =* 42	*n =* 80
	Limit certain vegetables and fruit	*n =* 3	-
	Improving vegetable and fruit quality	*n =* 2	*n =* 4
Grain Products	↑/specific serving number amounts	*n =* 1	*n =* 2
	Limit grain products (e.g., bread) or specific grain products	*n =* 7	*n =* 9
	↑ whole grains/try new grains	*n =* 5	*n =* 2
Milk and Alternatives	↑/specific serving number amount	*n =* 2	*n =* 1
	Limit milk and alternatives	*n =* 8	*n =* 3
	Choose healthier milk and alternatives (e.g., low fat products)	*n =* 3	-
Meat and Alternatives	↑/specific serving number amount	*n =* 1	*n =* 1
	Limit meat and alternatives (including limit red meat)	*n =* 6	*n =* 4
	Choose fish, lean meats, or meat alternatives	*n =* 12	*n =* 15
Limiting unhealthy foods/beverages and/or fat, cholesterol, sodium, and/or sugar in general		*n =* 140	*n =* 91
Alcohol		*n =* 22	*n =* 12
Water		*n =* 62	*n =* 53
Limit coffee and/or caffeine		*n =* 10	*n =* 2
Eat more/specific amounts of certain nutrients		*n =* 32	*n =* 57
Eat less/limit certain nutrients (except fat, sodium, and sugar)		*n =* 12	*n =* 31
Caloric/food intake amounts		*n =* 69	*n =* 28
Portion control		*n =* 10	*n =* 42
Self-monitor diet and/or ↑ awareness		*n =* 74	*n =* 19
Eating out less/Eat at home more		*n =* 8	*n =* 7
Evening/night time eating		*n =* 60	*n =* 10
Follow Canada’s Food Guide		*n =* 36	
Follow ﻿vegetarian/vegan diets		*n =* 12	
Follow fad diets		*n =* 15	
Gluten free (includes wheat free)		*n =* 21	
Follow other plans		*n =* 38	
Glycemic index		*n =* 4	
Clean eating		*n =* 15	
Supplements		*n =* 7	
Planning and preparing food		*n =* 29	*n =* 38
Goals describing when food is eaten or not eaten/eating patterns		*n =* 46	*n =* 31
Breakfast		*n =* 24	*n =* 1
Psychological aspects of eating		*n =* 31	*n =* 16
General eating improvements^a^		*n =* 71	*n =* 261
Other^b^		*n =* 6	*n =* 6

^a^Goals making reference to unspecific eating for a specific outcome (e.g., disease management) were coded as General Healthy Eating (lacks specificity) (*n =* 57 entries)

^b^Includes alternate/natural sweeteners, organic eating, adding/removing miscellaneous foods, avoiding allergens, eating as normal

Topics included as part of these goals not covered in the ready-made goals included stretching, core exercises, CrossFit®, tai chi, sports (e.g., hockey), step count goals (e.g., 10,000 steps/day), walking a certain distance by a specific date, walking dogs, being active during television commercials, high-intensity interval training, elliptical, exercise videos, activity tracking, and exergaming. Some goals included “or,” “/,” or “e.g.,” suggesting substitutions could take place.

Few goals (*n =* 40) for improving activity behaviours were directly linked to a reason (e.g., get active to lose weight). Weight management (usually weight loss) (*n =* 24) was the most common reason. Other reasons included improving: fitness (*n =* 8), body composition (*n =* 7), diabetes/blood sugar control (*n =* 2), blood cholesterol (*n =* 1), and health/energy levels (*n =* 1). Importantly, *n =* 26 of these goals had unspecific activity type and duration.

#### Nutrition behaviour change goals

Overall, *n =* 1,349 write your own goal statements were solely nutrition behaviour change goals; an additional *n =* 219 statements encompassed both nutrition behaviour change and other goal types without forming a direct link. This section will discuss nutrition behaviour change goals included in these *n =* 1,568 statements set by *n =* 1,179 users.

Table 4 classifies nutrition behaviour change goals by topic and specificity. The most common goals were to make general healthy eating improvements (*n =* 71 statements were more specific; *n =* 261 were less specific, e.g., re amount), followed by limiting unhealthy foods/beverages and/or fat, cholesterol, sodium, and/or sugar in general (*n =* 140 more specific; *n =* 91 less specific), increasing/eating specific amounts of vegetables and fruit (*n =* 42 more specific; *n =* 80 less specific) and drinking water (*n =* 62 more specific, *n =* 53 less specific). Importantly, some goals especially those for limiting unhealthy foods/beverages and/or fat, cholesterol, sodium, and/or sugar in general were potentially unrealistic (e.g., no sugar). Some goals also specified wanting to learn about nutrition.

These write your own goals generally had similar topics to ready-made goals. Additional topics included: limiting night time eating, decreasing alcohol, and psychological aspects of eating. Several goals also focused on specific nutrients (e.g., protein, carbohydrates), and meeting specific caloric targets. In general, these goals followed healthy eating recommendations; however, this was not true for all goals (e.g., follow fad diets).

Few nutrition goals (*n =* 139) were directly linked to a reason for improving behaviours. When reason(s) were directly specified, weight management was the most popular (*n =* 75) (note: only *n =* 2 goals were for weight gain), followed by diabetes (*n =* 22), other conditions (e.g., cholesterol/heart healthy, surgery preparation, arthritis, cancer, uric acid, acid reflux, irritable bowel syndrome, gas, bloating, uric acid, hypertension, anemia, kidney stones, pancreatitis, arthritis) (*n =* 22), sports nutrition/improving fitness (*n =* 12), body composition (*n =* 8), conception, pregnancy, and/or breastfeeding (*n =* 8), and health, energy or sleep improvements (*n =* 5).

### Association between goal topic area and user demographics

Numerous statistically significant independent associations were found between self-reported demographic variables (i.e., sex, age, BMI, activity level, pregnancy status, and breastfeeding status) and goal topic area. These are outlined in Table 5 and the odds ratios for each independent variable for each goal topic area are presented in Additional file 3. Because there are too many findings to describe in the results section, only a few notable findings for age, sex, and BMI are discussed here.

**Table 5:** Association between user demographics and types of goals set with the eaTracker® My Goals feature

Sex	Males were significantly (*p <* 0.05) less likely compared to females to set goals related to:	Males were more significantly (*p <* 0.05) more likely compared to females to set goals related to:
	• Activity (RM/WYO) • Vegetables and fruit (RM/WYO) • Planning/preparing food (RM/WYO) • When/where foods eaten (RM/WYO) • Evening/night time eating (WYO) • Psychological aspects of eating (WYO) • Generic healthy eating (WYO) • Weight loss (WYO) • Weight management/maintenance (WYO) • *Be healthier* (WYO) • *Eat more of certain nutrients* (WYO)	• Grain products (RM/WYO) • Breakfast (RM/WYO) • Weight gain (WYO) • Body composition (WYO) • *Alcohol* (WYO)
Age (with increasing age)	Significantly (*p <* 05) less likely to set goals related to:	Significantly (*p <* 0.05) more likely to set goals related to:
	• Activity (RM/WYO) • Breakfast (RM/WYO) • Eating out (RM/WYO) • Body composition (WYO) • *Planning and preparing food* (RM/WYO)	• Grain products (RM/WYO) • Meat and alternatives (RM/WYO) • Portion control (RM/WYO) • Calorie/food intake amounts (RM/WYO) • When/where foods eaten (RM/WYO) • Evening/night time eating (WYO) • Alcohol (WYO) • Eat less of certain nutrients (WYO) • Generic healthy eating (WYO) • Weight loss (WYO) • Weight management/maintenance (WYO) • Disease management (WYO) • Be healthier (WYO)
BMI (with increasing BMI)	Significantly (*p <* 0.05) less likely to set goals related to:	Significantly (*p <* 0.05) more likely to set goals related to:
	• Vegetables/fruit (RM/WYO) • Milk and alternatives (RM/WYO) • Meat and alternatives (RM/WYO) • Eating more of certain nutrients (WYO) • Weight gain (WYO) • Weight management/maintenance (WYO) • Body composition (WYO) • *Grain products* (RM/WYO)	• Activity (RM/WYO) • Breakfast (RM/WYO) • Portion control (RM/WYO) • Eating out (RM/WYO) • When/where food eaten (RM/WYO) • Self-monitoring (WYO) • Weight loss (WYO)
Activity	Significantly (*p <* 0.05) related to goals for: activity (RM/WYO), vegetables and fruit (RM/WYO), meat and alternatives (RM/WYO), breakfast (RM/WYO), water (RM/WYO), evening/night time eating (WYO), follow Canada’s Food Guide (RM/WYO), generic healthy eating (WYO), weight management/maintenance (WYO), body composition (WYO), disease management (WYO), improve fitness (WYO), *fat, sodium, sugar, unhealthy foods* (RM/WYO)*, when/where food is eaten* (RM/WYO)*, eating out* (RM/WYO), *eat more of certain nutrients* (WYO), *weight loss* (WYO)	
Pregnant (P)/ Breastfeeding (B)	Significantly (*p <* 0.05) less likely to set goals related to:	Significantly (*p <* 0.05) more likely to set goals related to:
	• Activity (RM/WYO) (P only) • Weight loss (WYO) (P only) • *When/where food eaten* (RM/WYO) (P only)	• Milk and alternatives (RM/WYO) (P only) • Evening/night time eating (WYO) (B only) • *Planning/Preparing food* (RM/WYO) (P only)

Ready-made and write your own goals were grouped together into fewer topic areas

RM/WYO: ready-made and write your own goals grouped together; WYO: write your own goals only

Analysis encompassed multivariate binary logistic regression with goal type as the dependent variable (set goal type vs. did not set goal type). Sex, age, BMI, pregnancy, breastfeeding, and self-reported activity were independent variables. Analysis was conducted with SPSS version 23 (IBM Corp, Armonk, NY). Analysis included *n =* 7,979 users as it excluded *n =* 88 users who had either an implausible BMI or an unspecified activity level

Goals in *italics* were borderline statistically significant (p values between 0.05 and 0.1)

For sex, some notable findings were that compared with males, females were statistically significantly (*p <* 0.05) more likely to set the following goals: activity (28.0 % (1,881/6,717) of females vs. 17.9 % (242/1,350) of males), planning and preparing food (18.5 % (1,243/6,717) of females vs. 14.5 % (196/1,350) of males), vegetables and fruit (28.3 % (1,903/6,717) of females vs. 21.6 % (292/1,350) of males), when and where foods are eaten (22.9 % (1,540/6,717) of females vs. 18.7 % (253/1,350) of males), evening/night time eating (0.9 % (60/6,717) of females vs. 0.3 % (4/1,350) males), psychological aspects of eating (0.6 % (41/6,717) of females vs. 0.1 % (1/1,350) of males), weight loss (16.9 % (1,138/6,717) of females vs. 12.7 % (172/1,350) of males), weight management/maintenance (1.9 % (130/6,717) of females vs. 0.8 % (11/1,350) of males). Compared to females, males were statistically significantly (*p <* 0.05) more likely to set goals related to grain products (6.4 % (87/1,350) of males vs. 4.6 % (307/6,717) of females), breakfast (9.0 % (121/1,350) of males vs. 5.7 % (383/6,717) of females), weight gain (2.3 % (31/1,350) of males vs. 0.3 % (20/6,717) of females), and body composition (3.9 % (53/1,350) of males vs. 1.9 % (127/6,717) of females).

For age, users who set the following goals were significantly younger (*p <* 0.05) than those who did not: eating out (38.7 ± 13.4 years of age for yes vs. 41.2 ± 15.1 years of age for no), breakfast (37.5 ± 14.3 years of age for yes vs. 41.4 ± 15.0 years of age for no), and body composition (33.5 ± 13.4 years of age for yes vs. 41.3 ± 15.0 years of age for no). Users who set the following goals were significantly older (*p <* 0.05) than those who did not: grain products (43.2 ± 15.6 years of age for yes vs. 41.0 ± 15.0 years of age for no), meat and alternatives (42.9 ± 15.5 years of age for yes vs. 41.0 ± 15.0 years of age for no), when/where foods eaten (42.6 ± 14.7 years of age for yes vs. 40.7 ± 15.1 years of age for no), portion control (44.2 ± 14.4 years of age for yes vs. 40.4 ± 15.1 years of age for no), calorie/food intake amounts (44.3 ± 15.0 years of age for yes vs. 41.0 ± 15.0 years of age for no), alcohol (51.3 ± 12.3 years of age for yes vs. 41.1 ± 15.0 years of age for no), eat less of certain nutrients (47.5 ± 16.0 years of age for yes vs. 41.1 ± 15.0 years of age for no), evening/night time eating (46.0 ± 15.1 years of age for yes vs. 41.1 ± 15.0 years of age for no), weight loss (44.6 ± 14.9 years of age for yes vs. 40.5 ± 14.9 years of age for no), be healthier (45.5 ± 15.1 years of age for yes vs. 41.1 ± 15.0 years of age for no), and disease prevention/management (53.4 ± 12.2 years of age for yes vs. 40.9 ± 14.9 years of age for no).

For BMI, users who had set the following goals had a statistically significantly (*p <* 0.05) lower BMI compared to those who did not: milk and alternatives (26.9 ± 7.0kg/m^2^ for yes vs. 28.8 ± 7.6kg/m^2^ for no), meat and alternatives (27.4 ± 7.1kg/m^2^ for yes vs. 28.9 ± 7.6kg/m^2^ for no), eat more of certain nutrients (26.1 ± 8.4kg/m^2^ for yes vs. 28.8 ± 7.6kg/m^2^ for no), weight gain (20.7 ± 2.8kg/m^2^ for yes vs. 28.9 ± 7.6kg/m^2^ for no), and body composition (24.8 ± 5.0kg/m^2^ for yes vs. 28.9 ± 7.6kg/m^2^ for no). Users who had set the following goals had a statistically significantly (*p <* 0.05) higher BMI compared to those who did not: activity (29.8 ± 7.9kg/m^2^ for yes vs. 28.5 ± 7.4kg/m^2^ for no), eating out (30.2 ± 7.3kg/m^2^ for yes vs. 28.7 ± 7.6kg/m^2^ for no), when/where foods are eaten (29.6 ± 7.4kg/m^2^ for yes vs. 28.6 ± 7.6kg/m^2^ for no), portion control (30.0 ± 7.0kg/m^2^ for yes vs. 28.5 ± 7.7kg/m^2^ for no), self-monitoring (32.4 ± 9.9kg/m^2^ vs. 28.8 ± 7.5kg/m^2^), and weight loss (30.4 ± 7.2kg/m^2^ for yes vs. 28.5 ± 7.6kg/m^2^ for no).

### Association between demographic variables and My goals tracker use

Multivariate binary logistic regression revealed that age and physical activity level were statistically significantly related to use of the My Goals Tracker (i.e., tracked ≥1 goal vs. did not track any goals as the outcome). This analysis is presented in Table 6.

**Table 6:** Association between eaTracker® My Goals user demographics and My Goals Tracker use

	Adjusted OR [95 % CI]
**Sex**	
Female	1.0
Male	0.996 [0.796–1.245]
**Age**	**1.007** **[1.001–1.013]**
**BMI**	1.006 [0.994–1.017]
**Physical Activity Level**	
Sedentary	1.0
Low Active	**0.781** **[0.624–0.977]**
Active	**0.762** **[0.588–0.986]**
Very Active	0.822 [0.517–1.305]
**Pregnant**	
No	1.0
Yes	0.765 [0.353–1.658]
**Breastfeeding**	
No	1.0
Yes	1.494 [0.848–2.633]

Multivariate binary logistic regression with ≥1 goal tracked (yes or no) as the outcome

Bold values are significant (*p <* 0.05)

## Discussion

To our knowledge, this is the first study that has retrospectively analyzed data from a large group of individuals who accessed a publicly available website-based nutrition and physical activity behaviour change goal setting and tracking feature not for the purposes of a research trial. Overall, eaTracker® users showed interest in the My Goals feature (i.e., *n =* 16,511 goal statements were written by *n =* 8,067 users in ~17 months which was ~27.2 % of all users from Ontario and Alberta). This interest suggests further research on these types of features as well as how to strengthen them for use as part of publicly available electronic nutrition and physical activity behaviour change tools is warranted.

Overall, there were a higher proportion of My Goals users who self-reported overweight or obesity compared to the general population of Canadians from approximately the same time period [44]. However, users generally shared similar demographics to those reported in other studies examining use of public weight management websites (e.g., females <50 years of age with overweight and/or obesity) [45–48]. Although reasons for using the My Goals feature were not directly captured, there was evidence that users often set weight management goals; this finding is not surprising given the high prevalence of overweight and obesity in Canadian adults [3]. However, other types of goals were also set (e.g., disease management, weight gain) which suggests that publicly available tools should be able to accommodate varied needs and goal topics.

Ready-made goals were very popular amongst users of the My Goals feature; in fact ~75 % of all goals set with the My Goals feature by users in this dataset were ready-made goals. This finding is promising in the sense that users appeared to be interested as a whole in this type of goal setting which helps them to set SMART behaviour based goals which follow Canadian nutrition [43] and physical activity guidelines [49]. These findings confirm that ready-made goals should continue to be included as part of the My Goals feature as well as suggest that these types of goals should also be considered for inclusion in future tools looking to incorporate goal setting and tracking features.

Many users capitalized on the opportunity to write their own goals with the My Goals feature (i.e., *n =* 2,587 users created *n =* 4,062 write your own goal statements). Although several users took advantage of this opportunity, there were some issues that were uncovered from the analysis of these goals that are useful for future advancement the My Goals feature and the development other similar types of tools. For example, several write your own goals in this dataset did not adhere to health guidelines (e.g., fad diets, weight loss amounts >1kg/week, very low calorie diets), were non-specific (e.g., missing amount), possibly unrealistic (e.g., no sugar) and were poor quality according to Goal Setting Theory and SMART criteria. Write your own goals also were commonly focused on longer term (distal) goals (e.g., lose weight) versus those that are behaviour based. In general, many goals would also not generally follow standards about how goals are to be used for behavioural therapy for obesity [11, 12] and within the 5As for obesity management [14, 15] (e.g., focus on small changes in behaviour). Some users also appeared to have some difficulties choosing appropriate frequencies for their goals (e.g., lose 5kg; frequency: daily); it is unclear whether this finding is due to a lack of understanding how to use the frequency tool in the My Goals feature or about what is realistic or both. These findings regarding poor quality goals are not entirely surprising based on previous work that has provided data on the types of goals individuals set. Findings from these studies have found that individuals may set: a) weight loss goals that do not match professional recommendations or want to lose weight quickly via dieting [50, 51], and b) poor quality goals in general (e.g., non-specific, vague, broad, not behaviour based) [27, 28]. In addition, instructions to on how to write SMART goals available for Ontario users only may not have been used often as upon scanning the write your own goals, poor quality goals were roughly proportionately as common in Ontario users as Alberta users who were not provided with instructions. Lack of use of instructions on goal writing may have also occurred with an online epilepsy management goal setting program [41]. Although providing individuals the option to write in their own goals is important, resources and checkpoints (preferably automated and beyond just providing instructions) are needed to ensure quality goals are set that adhere both to guidelines (e.g., SMART, safe), and are also appropriate for the individual user when health professional support is unavailable. Moreover, having places in the feature where users can set outcome goals as well as behaviour based goals is important and may help them to understand that smaller behaviour based goals will help them reach a long term (distal) goal (e.g., weight management) [15].

Although other studies have reported on topic areas of interest to users of these types of tools (e.g., fiber, fat/sugar) [34, 38], this is really the first study to comprehensively report information on the types of goals set as well as the associations between demographic variables and types of goals set. This information is helpful both to create more relevant ready-made goals for users of the My Goals feature as well as for the broader research and professional community looking to develop or modify similar types of tools.

Previous work has shown that engagement with website-based tools for nutrition and physical activity behaviour change is associated with positive outcomes (e.g., weight loss) [46, 48, 52, 53]. However, this study and previous studies [45, 48] have found that non-use of website features is common. In fact, this phenomena is so common in electronic health interventions that Eysenbach [54] proposed the “Law of Attrition” which suggests that there are high levels of intervention non-use (non-use attrition) and/or dropouts (dropout attrition) in electronic self-help interventions; one reason for this phenomena is that electronic intervention use dosages are more user chosen and are not absolutely essential for health unlike drug trials with prescribed doses and possibly more immediate effects [54]. Although this study found that demographic and behavioural variables (specifically age and activity level) were associated with goal tracking which is similar to previous studies which have found linkages between various demographic and behavioural variables (e.g., age, sex, diet, health behaviours) and website use [47, 55, 56], these variables likely do not explain the whole story of non-use. Factors including user characteristics (e.g., mood, lack of self-discipline, health problems, motivation) [54, 57, 58], innovation characteristics [54, 57], limited time [59, 60], and challenges with self-monitoring (including website-based self-monitoring) in general [61, 62] (which could affect overall eaTracker® website use) are all factors that have been previously suggested to affect use of electronic tools which could have also affected My Goals use. A qualitative study on wearables (e.g., Fitbit® (Fitbit, San Francisco, CA)) [63] found that some users like virtual rewards for goal achievement which is something that the My Goals feature did not provide and could be another factor that contributed to non-use. In addition, another possible reason that users may not have followed through with their goals is that the My Goals feature does not have any online component that helps users work through facilitators and barriers that may affect their goal achievement (although Ontario users would have had the option to contact an EatRight Ontario contact centre dietitian for this support and the motivational messages do provide tips) which is an commonly used technique in behavioural management for obesity [11]. Moreover, there were no tools to help guide individuals on which goals should be chosen for their given situation. Because this type of personalized support was not present, it is possible users may have given up on their goals more quickly. Incorporating online tools to provide this type of assistance in future iterations may help users to be more adherent. Qualitative research on users of the My Goals feature or similar tools may also provide insight into non-use and desired features which may help to increase adherence to use of such tools in the future.

This study has some important strengths. First, it is only one of a handful of studies that have examined use of publicly available websites for nutrition and physical activity behaviour change not used for the purpose of a research study; most studies are conducted in research trial settings with motivated participants. Second, to our knowledge, this is also the first study to assess use of a nutrition and physical activity behaviour goal setting and tracking tool outside of a research trial setting. Third, this study also linked user demographics to the types of goals set in users of a publicly available tool which has never been done before. Although this study has some important strengths, there are also some limitations that should be noted. First, demographic data, including height and weight, were self-reported, and data such as socioeconomic status, and ethnicity were not captured. Second, a sense of user motivation for behaviour change could not be obtained; it was unclear whether users were just trialing the website or were really interested in changing their behaviours. Third, when assessing goal quality, a sense of whether goals were realistic and appropriate for individual users could not be obtained. Fourth, this study did not capture how use of My Goals compares with use of other aspects of the eaTracker® website, and as well how these Alberta and Ontario My Goals users compare with the larger Canadian population of My Goals users. Lastly, we were not able to obtain solid data on whether individuals met their goals with this tool because of high rates of non-use of the tracker. This is a well described problem with electronic health tools in general [54] and qualitative research on user experiences and perceptions with the My Goals feature would provide some needed insight into reasons for the low rate of use of this tool.

## Conclusions

Substantial interest exists in the My Goals feature as part of the broader eaTracker® nutrition and physical activity self-monitoring website, however, there were high rates of non-use after initial goal setting. In addition, many of the write your own goals were poor quality and may not set users up for success. With the popularity of website-based tools to facilitate nutrition and physical activity behaviour change, goal setting, and limited access to professional support for assistance, website-based tools represent an important future direction to assist individuals with use of goals for nutrition and physical activity behaviour change. Future research needs to determine how to help individuals write better quality goals using website-based tools (instructions may not be enough) and how to help users to follow-up with their goals; qualitative research on users of these tools may provide important insight to help move this area forward.

## Additional files

Additional file 1: Sample eaTracker® My Goals feature ready-made goals. (PDF 92 kb) Additional file 2: eaTracker® My Goals feature and EatRight Ontario motivational messaging sample screenshots. (PDF 847 kb) Additional file 3: Odds ratios from multivariate binary logistic regression analysis assessing the association between eaTracker® My Goals feature user demographics and types of goals set with the My Goals feature. (PDF 260 kb)

## Abbreviations

BMI: Body mass index
SMART: Specific, Measurable, Attainable, Realistic, Time-related
