# Supplementary material for: A retrospective analysis of real-world use of the eaTracker® My Goals website by adults from Ontario and Alberta, Canada
Source: BMC Public Health. 2016 Sep 15;16:978. doi: 10.1186/s12889-016-3640-6 (PMC5024431; doi:10.1186/s12889-016-3640-6)
Supplement: Additional file 1: — Sample eaTracker® My Goals feature ready-made goals. (PDF 92 kb) [file 12889_2016_3640_MOESM1_ESM.pdf]

Additional File 1: Sample eaTracker® My Goals feature ready-made goals

Categories n=13; n=87 total goals (only sample goals are shown)

Category: Choosing more vegetables and fruit (n=7 goals; n=3 sample goals shown)
1) Eat a dark green vegetable every day this week (e.g. spinach, broccoli, Romaine lettuce, bok choy, swiss chard).
2) Eat at least 7 servings of vegetables and fruit every day this week. 1 serving is 1 piece of fruit, 1/2 a cup of fruit or vegetables or 1 cup of leafy vegetables.
3) Make half my plate vegetables at lunch or dinner every day this week.

Category: Choosing healthier grain products (n=3 goals; n=1 sample goal shown)
1) Eat whole grain bread or cereal 3 times this week.

Category: Choosing healthier milk and alternatives (n=3 goals; n=1 sample goal shown)
1) Eat lower fat yogurt (less than 2% Milk Fat or M.F.) instead of full fat yogurt this week.

Category: Choosing healthier meat and alternatives (n=6 goals; n=3 sample goals shown)
1) Buy and use lean or extra lean ground beef instead of regular ground beef this week.
2) Eat one meal with legumes (e.g. beans, peas or lentils) this week.
3) Have fish (e.g. salmon, trout, sole, halibut) 2 times this week.

Category: Choosing healthier beverages (n=6 goals; n=2 sample goals shown)
1) Replace pop and juice with drinks like water or milk this week.
2) Drink one extra glass of water every day this week.

Category: Eating a healthy breakfast (n=5 goals; n=2 sample goals shown)
1) Avoid baked goods (e.g. donuts, pastries, muffins) at breakfast this week.
2) Eat a high fibre cereal (4g per serving) for breakfast 3 times this week.

Category: Eating a healthy lunch (n=6 goals; n=2 sample goals shown)
1) Eat two vegetarian lunches this week.
2) Replace deli meat with leftover cooked chicken or turkey for lunch this week.

Category: Eating a healthy dinner (n=4 goals; n=2 sample goals shown)
1) Eat dinner with 3 of the 4 food groups every day this week (e.g. brown rice, steamed vegetables and salmon).
2) Prepare and eat dinner at home at least 3 times this week.

Category: Getting more fibre (n=4 goals; n=2 sample goals shown)
1) Eat brown rice or whole wheat pasta instead of white rice or white pasta this week.
2) Eat fruits and vegetables with the skin on them (e.g. apples, pears, potatoes) this week.

Category: Eating less fat, sodium or sugar (n=11 goals; n=2 sample goals shown)
1) Avoid all fried foods this week.
2) Compare the Nutrition Facts Table on food labels and choose products that have less fat and sodium when grocery shopping this week.

Category: Managing your weight (n=9 goals; n=3 sample goals shown)
1) Avoid second helpings during meals and snacks every day this week.
2) Avoid snacking while reading, using the computer or watching TV every day this week.
3) Do not skip meals any day this week.

Category: Planning and preparing food (n=5 goals; n=2 sample goals shown)
1) Make one new healthy recipe for dinner this week.
2) Make a menu plan and shopping list and use it this week.

Category: Getting active (n=18 goals; n=3 sample goals shown)
1) Be physically active for 20 minutes 2 times this week (e.g. brisk walking, jogging, bike riding, dancing, swimming).
2) Do an outdoor family activity with the kids this weekend (e.g. go for a hike, play at the park, play tag in the backyard).
3) Walk briskly for 10 minutes every day this week.

© 2016 Dietitians of Canada www.eaTracker.ca
