# Supplementary material for: A retrospective analysis of real-world use of the eaTracker® My Goals website by adults from Ontario and Alberta, Canada
Source: BMC Public Health. 2016 Sep 15;16:978. doi: 10.1186/s12889-016-3640-6 (PMC5024431; doi:10.1186/s12889-016-3640-6)
Supplement: Additional file 2: — eaTracker® My Goals feature and EatRight Ontario motivational messaging sample screenshots. (PDF 847 kb) [file 12889_2016_3640_MOESM2_ESM.pdf]

Additional File 2: eaTracker® My Goals feature and EatRight Ontario motivational messaging sample screenshots

Main eaTracker® login page (http://www.eaTracker.ca/)

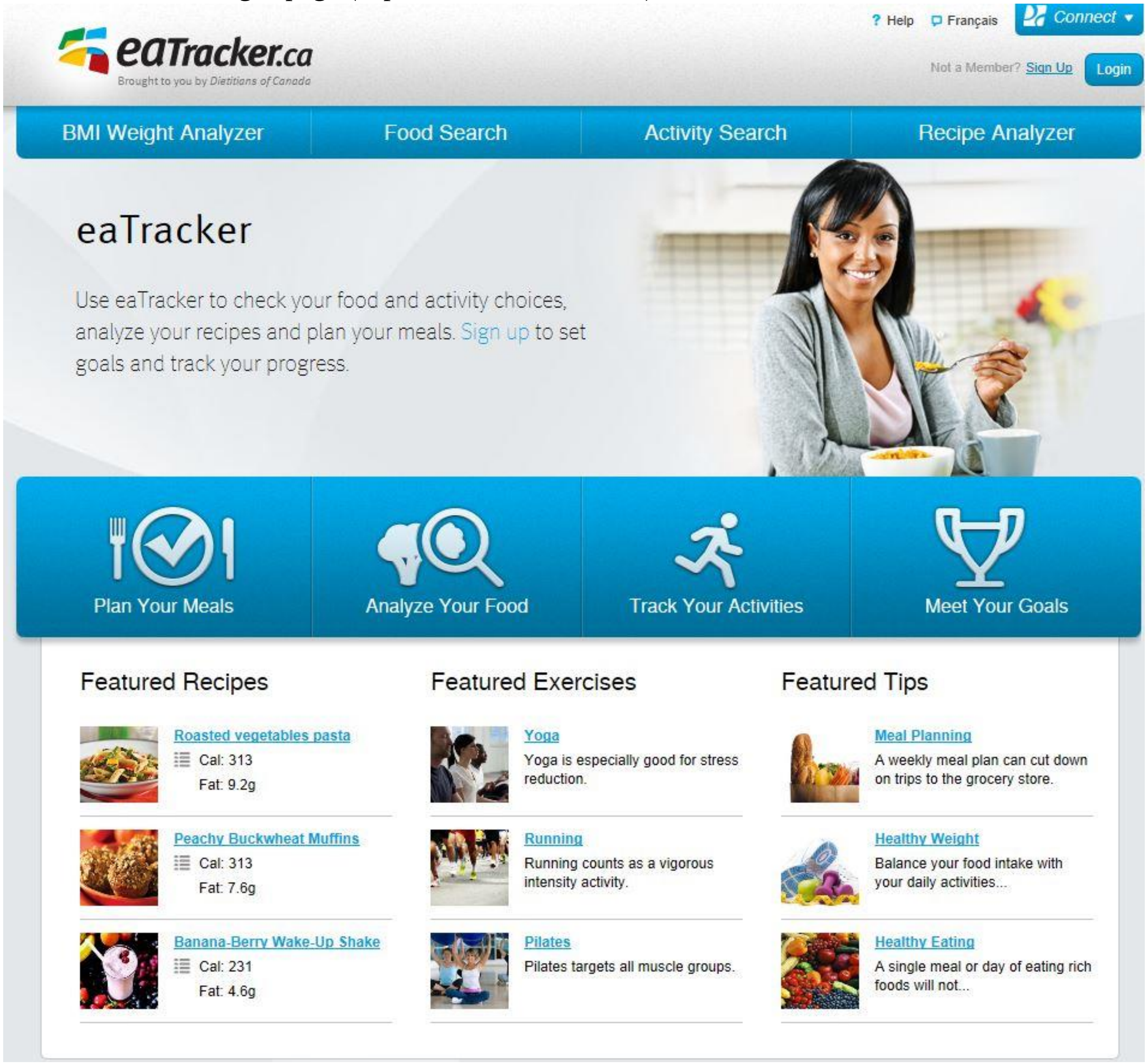

© 2016 Dietitians of Canada www.eaTracker.ca

Main Goal Setting Page

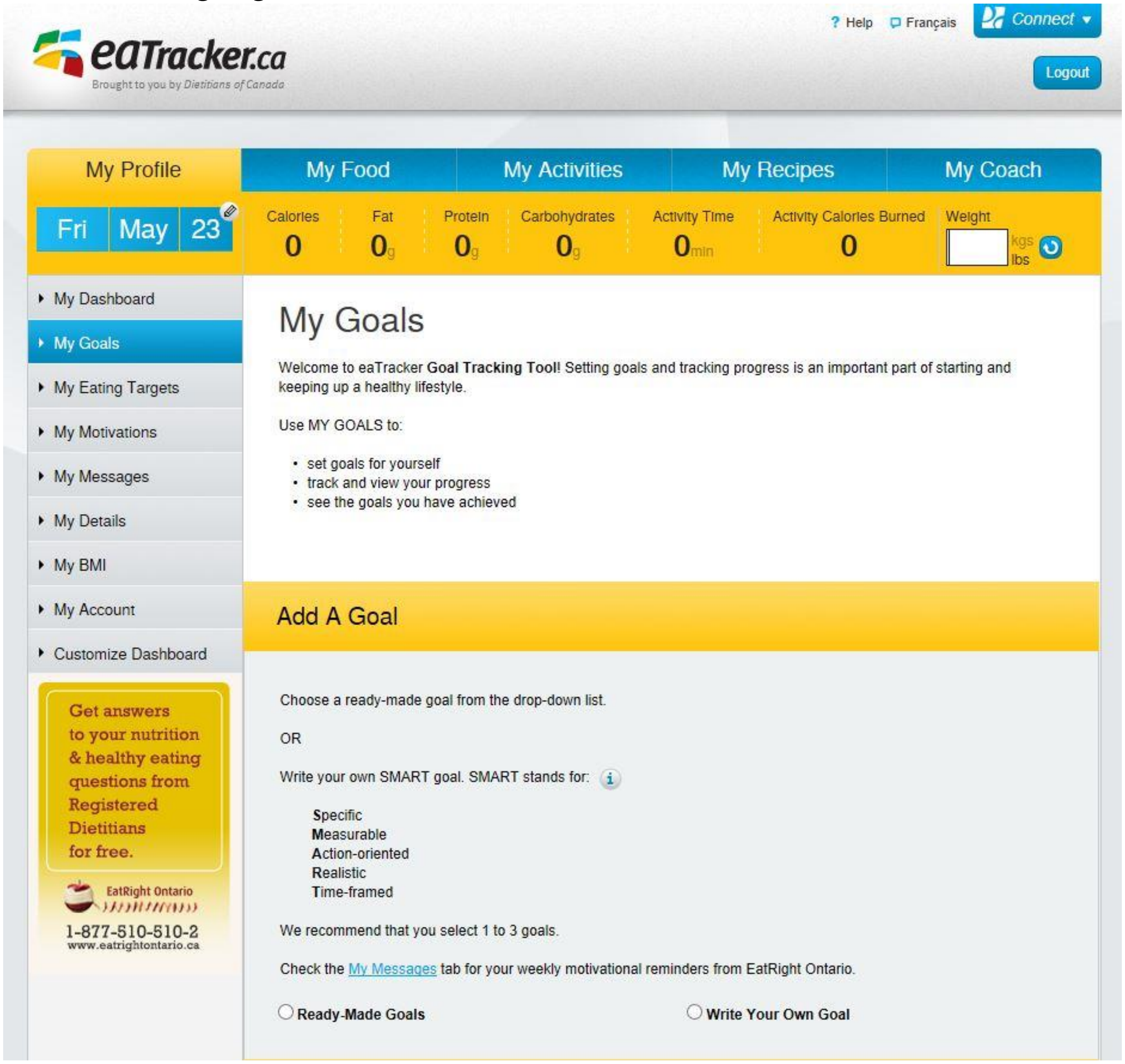

© 2016 Dietitians of Canada www.eaTracker.ca

Ready-Made Goal Setting

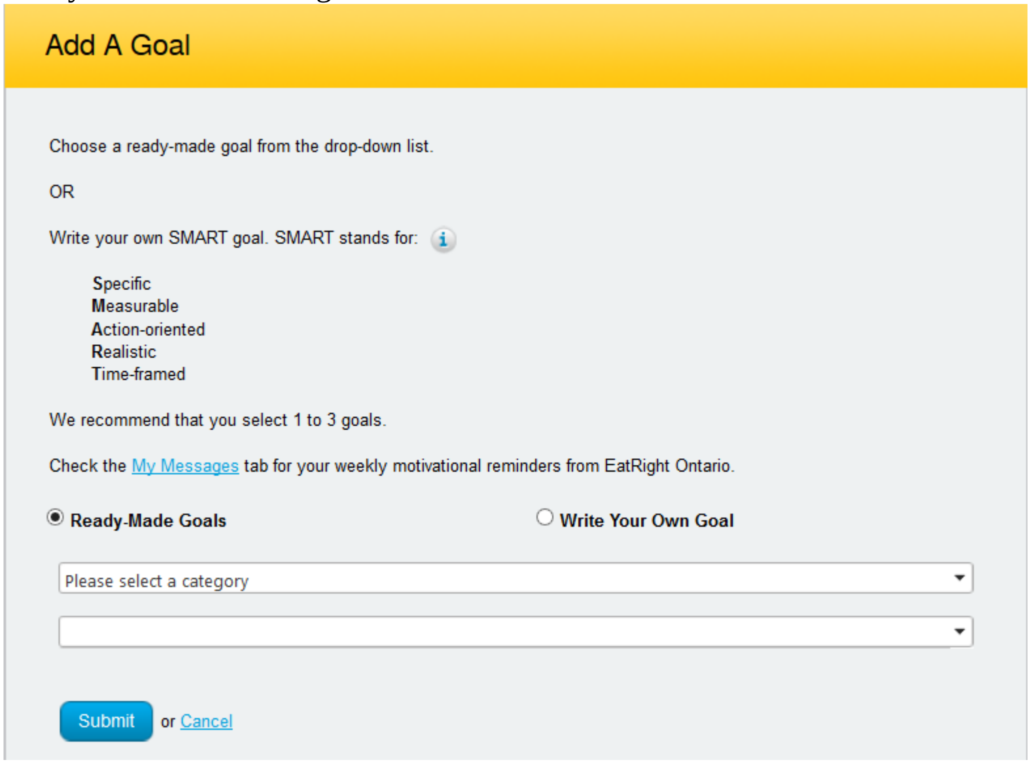

Add A Goal

Choose a ready-made goal from the drop-down list.

OR

Write your own SMART goal. SMART stands for:

Specific
Measurable
Action-oriented
Realistic
Time-framed

We recommend that you select 1 to 3 goals.

Check the My Messages tab for your weekly motivational reminders from EatRight Ontario.

(●) **Ready-Made Goals** ( ) **Write Your Own Goal**

Please select a category

Submit or Cancel

© 2016 Dietitians of Canada www.eaTracker.ca

Write Your Own Goal Setting

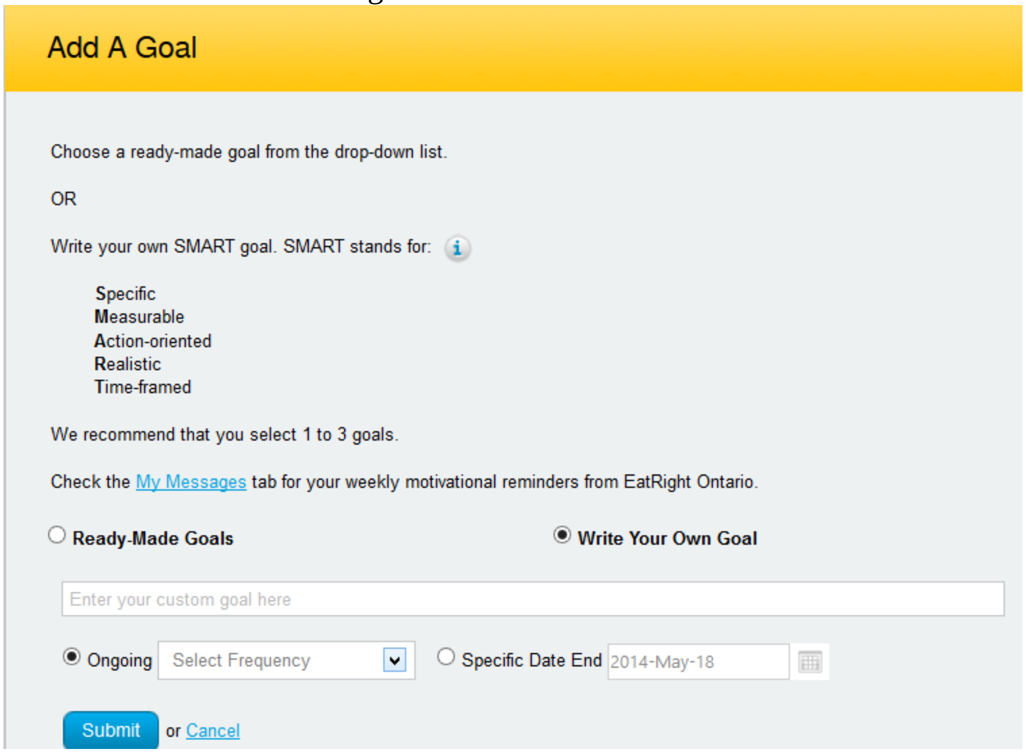

Add A Goal

Choose a ready-made goal from the drop-down list.

OR

Write your own SMART goal. SMART stands for:

Specific
Measurable
Action-oriented
Realistic
Time-framed

We recommend that you select 1 to 3 goals.

Check the My Messages tab for your weekly motivational reminders from EatRight Ontario.

( ) **Ready-Made Goals** (●) **Write Your Own Goal**

Enter your custom goal here

(●) Ongoing Select Frequency ( ) Specific Date End 2014-May-18

Submit or Cancel

© 2016 Dietitians of Canada www.eaTracker.ca

Goal Tracking

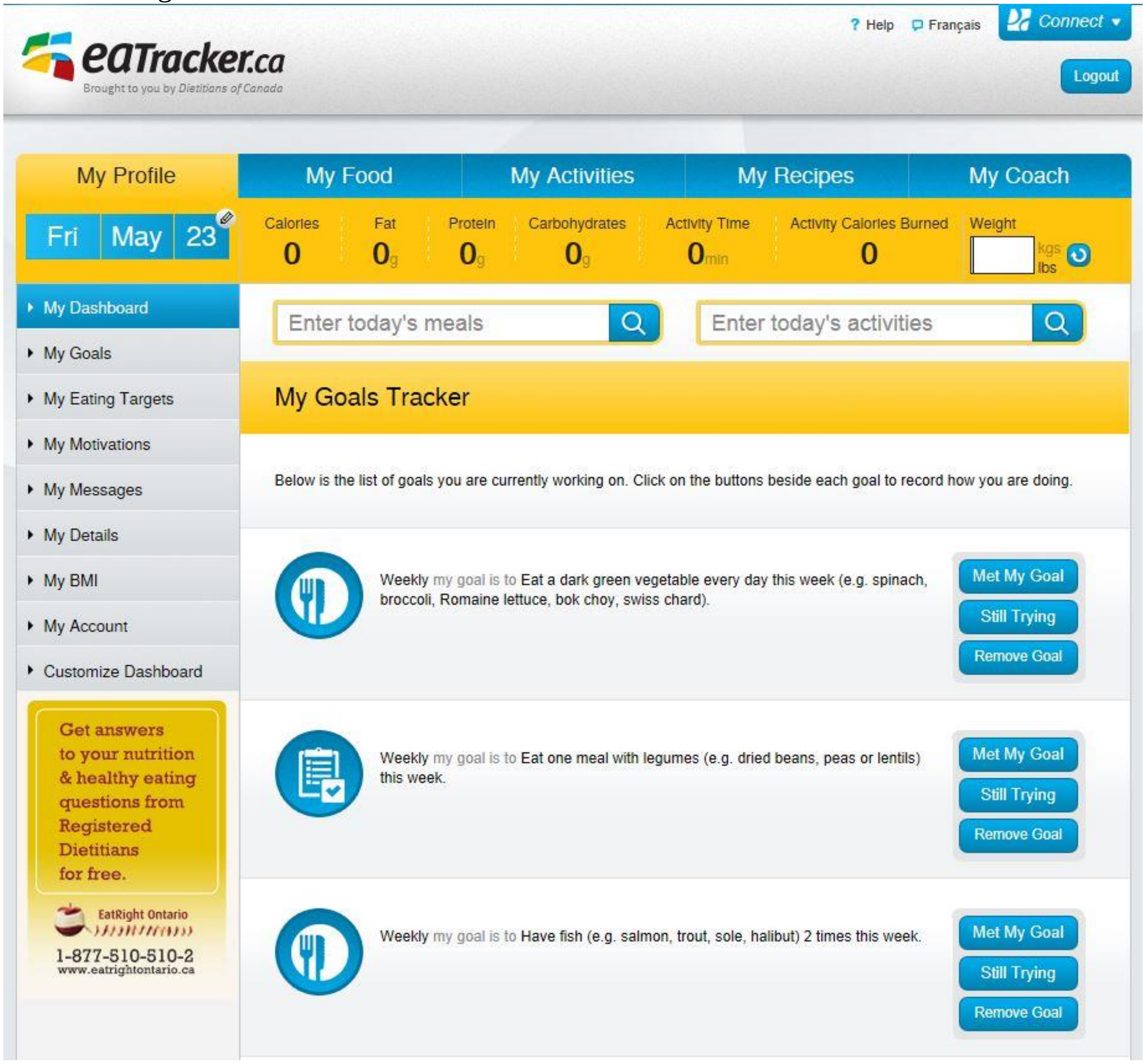

© 2016 Dietitians of Canada www.eaTracker.ca

Manage My Goals

Manage My Goals

Hide

See the goals you are working on below. View your progress.

Replace hot and cold sweetened coffee beverages with plain coffee made with 1 teaspoon of sugar and low fat milk.
Frequency : Weekly

Hide

Goal Completion History

Goal Date	Met My Goal	Still Trying
2014-May-20	•	

Eat one meal with legumes (e.g. dried beans, peas or lentils) this week.
Frequency : Weekly

History

© 2016 Dietitians of Canada www.eaTracker.ca

My Success

My Success

Hide

Congratulations on your goal activity. See the goals you have reached.

	Number of times you met your goal
Eat one meal with legumes (e.g. dried beans, peas or lentils) this week.	2

© 2016 Dietitians of Canada www.eaTracker.ca

EatRight Ontario motivational messaging

Sample email message

EatRight Ontario

eaTracker.ca
Brought to you by Dietitians of Canada

EatRight Ontario and eaTracker are here to help motivate you to reach your healthy eating and physical activity goals!

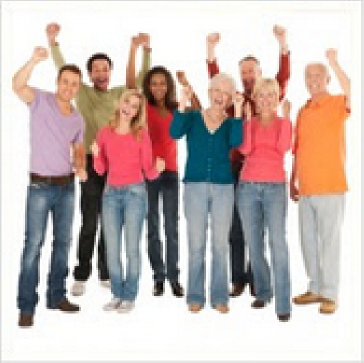

Your goal: Eat a dark green vegetable every day this week (e.g. spinach, broccoli, Romaine lettuce, bok choy, swiss chard).

Tip: To help you eat a dark green vegetable every day this week, add fresh or frozen chopped greens to vegetable soups, stews or chili.

Login to your www.eatracker.ca account and track your progress!

Quick Links

- EatRight Ontario
- My Menu Planner
- Email a Dietitian

If you have additional questions about nutrition or healthy eating please do not reply to this email instead:

- Call EatRight Ontario toll-free at 1-877-510-510-2
- Email a Dietitian from www.eatrightontario.ca

You are receiving this email because you have registered for EatRight Ontario's motivational messages and tips to help you meet your goal. These messages are also found in the MY MESSAGES section of www.eatracker.ca when you login to your account.

Click Here to stop receiving motivational messages about this goal.

To log into eaTracker, please click here

© 2016 Dietitians of Canada www.eaTracker.ca

eaTracker® message delivery – My Messages

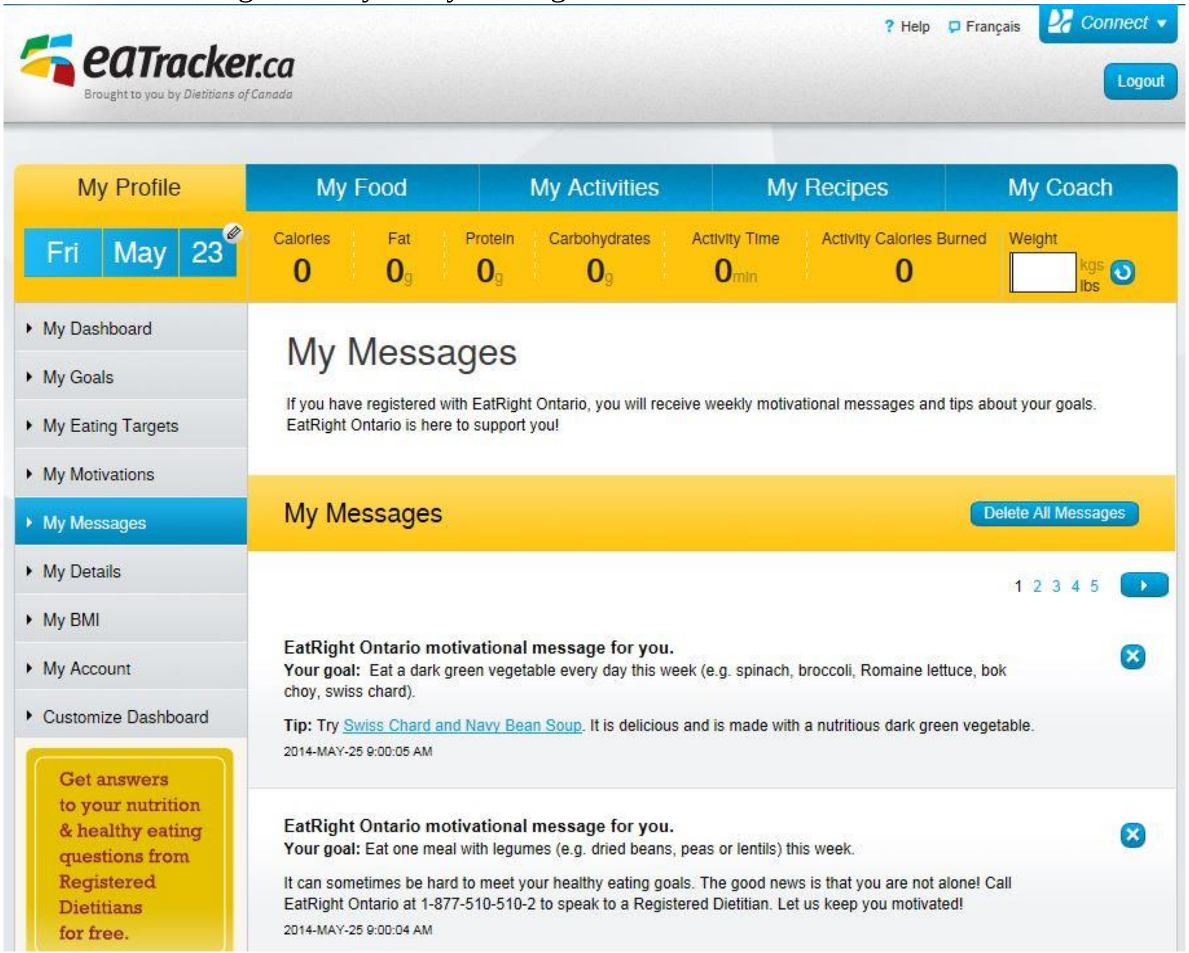

© 2016 Dietitians of Canada www.eaTracker.ca
