# Supplementary material for: A retrospective analysis of real-world use of the eaTracker® My Goals website by adults from Ontario and Alberta, Canada
Source: BMC Public Health. 2016 Sep 15;16:978. doi: 10.1186/s12889-016-3640-6 (PMC5024431; doi:10.1186/s12889-016-3640-6)
Supplement: Additional file 3: — Odds ratios from multivariate binary logistic regression analysis assessing the association between eaTracker® My Goals feature user demographics and types of goals set with the My Goals feature. (PDF 260 kb) [file 12889_2016_3640_MOESM3_ESM.pdf]

Additional File 3: Odds ratios from multivariate binary logistic regression analysis assessing the association between eaTracker® My Goals feature user demographics and types of goals set with the My Goals feature

	Activity	Vegetables and Fruit	Grain Products	Milk and Alternatives	Meat and Alternatives	Fat, Sodium, Sugar, Unhealthy Foods	Breakfast	Water
**Sex**								
Female	1.0	1.0	1.0	1.0	1.0	1.0	1.0	1.0
Male	**0.621 [0.533-0.723]**	**0.726 [0.629-0.836]**	**1.412 [1.098-1.817]**	1.192 [0.723-1.967]	1.028 [0.781-1.353]	0.983 [0.847-1.141]	**1.644 [1.319-2.048]**	0.885 [0.615-1.273]
**Age**	**0.995 [0.992-0.999]**	1.001 [0.997-1.004]	**1.013 [1.006-1.020]**	1.002 [0.988-1.016]	**1.015 [1.008-1.022]**	1.002 [0.998-1.006]	**0.979 [0.973-0.986]**	0.995 [0.986-1.004]
**BMI**	**1.011 [1.004-1.018]**	**0.993 [0.986-1.000]**	*0.984 [0.969-1.000]*	**0.961 [0.929-0.994]**	**0.952 [0.936-0.968]**	0.998 [0.990-1.006]	**1.014 [1.001-1.027]**	1.005 [0.987-1.023]
**Activity**								
Sedentary	1.0	1.0	1.0	1.0	1.0	1.0	1.0	1.0
Low Active	**0.809 [0.706-0.928]**	0.950 [0.824-1.096]	1.019 [0.747-1.390]	1.030 [0.555-1.913]	0.814 [0.616-1.077]	0.885 [0.756-1.035]	**0.776 [0.606-0.995]**	**0.690 [0.485-0.983]**
Active	**0.414 [0.351-0.489]**	*0.852 [0.725-1.001]*	1.103 [0.784-1.552]	1.164 [0.603-2.249]	**0.539 [0.387-0.749]**	0.869 [0.728-1.037]	**0.611 [0.457-0.816]**	0.827 [0.558-1.226]
Very Active	**0.215 [0.146-0.317]**	**0.660 [0.491-0.888]**	1.302 [0.761-2.226]	1.235 [0.453-3.365]	0.923 [0.559-1.525]	*0.746 [0.542-1.027]*	*0.653 [0.397-1.074]*	0.614 [0.281-1.337]
**Pregnant**								
No	1.0	1.0	1.0	1.0	1.0	1.0	1.0	1.0
Yes	**0.554 [0.346-0.888]**	1.350 [0.921-1.979]	0.788 [0.287-2.159]	**3.630 [1.427-9.235]**	1.109 [0.481-2.558]	1.134 [0.729-1.764]	1.243 [0.622-2.483]	1.431 [0.575-3.563]
**Breastfeeding**								
No	1.0	1.0	1.0	1.0	1.0	1.0	1.0	1.0
Yes	1.136 [0.781-1.652]	1.331 [0.926-1.912]	1.121 [0.487-2.577]	0.499 [0.068-3.668]	0.767 [0.310-1.899]	0.906 [0.583-1.410]	0.777 [0.359-1.682]	0.696 [0.218-2.217]

*odds ratio could not be estimated.
Bold values are statistically significant ($p<0.05$); *italics* are borderline statistically significant (p value between 0.05 and 0.1)

Analysis encompassed multivariate binary logistic regression with goal type as the dependent variable (set goal type, did not set goal type). Sex, age, BMI, pregnancy, breastfeeding, and self-reported activity were independent variables. Analysis was conducted with SPSS version 23 (IBM corp, Armonk, NY). Analysis included n=7,979 users as it excluded n=88 users who had either an implausible BMI or an unspecified activity level.

	Planning and Preparing Food	Portion Control	Calorie/Food Intake Amounts	Eating Out	When/where foods are eaten	Evening/night time eating	Psychological Aspects of Eating	Alcohol
**Sex**								
Female	1.0	1.0	1.0	1.0	1.0	1.0	1.0	1.0
Male	**0.753 [0.638-0.889]**	0.989 [0.847-1.155]	0.934 [0.634-1.376]	0.971 [0.722-1.306]	**0.780 [0.671-0.907]**	**0.335 [0.121-0.929]**	**0.121 [0.017-0.884]**	*2.027 [0.931-4.414]*
**Age**	*0.996 [0.992-1.000]*	**1.014 [1.010-1.018]**	**1.015 [1.005-1.025]**	**0.982 [0.974-0.990]**	**1.005 [1.002-1.009]**	**1.024 [1.005-1.043]**	0.999 [0.977-1.021]	**1.052 [1.026-1.078]**
**BMI**	1.003 [0.995-1.011]	**1.016 [1.008-1.024]**	0.997 [0.976-1.018]	**1.029 [1.015-1.043]**	**1.014 [1.007-1.022]**	1.005 [0.970-1.041]	0.994 [0.949-1.041]	0.961 [0.908-1.018]
**Activity**								
Sedentary	1.0	1.0	1.0	1.0	1.0	1.0	1.0	1.0
Low Active	0.899 [0.762-1.060]	1.080 [0.917-1.273]	0.957 [0.632-1.449]	0.927 [0.691-1.246]	*1.143 [0.978-1.335]*	**0.511 [0.265-0.985]**	0.933 [0.367-2.367]	1.553 [0.528-4.565]
Active	0.923 [0.766-1.111]	1.044 [0.866-1.259]	1.057 [0.665-1.681]	*0.739 [0.522-1.048]*	1.131 [0.948-1.348]	0.786 [0.378-1.637]	1.380 [0.512-3.722]	0.615 [0.159-2.376]
Very Active	0.943 [0.683-1.302]	0.808 [0.566-1.155]	1.457 [0.705-3.011]	*0.525 [0.255-1.084]*	0.760 [0.542-1.066]	1.461 [0.456-4.679]	0.706 [0.082-6.103]	1.832 [0.315-10.667]
**Pregnant**								
No	1.0	1.0	1.0	1.0	1.0	-*	1.0	-*
Yes	*1.432 [0.940-2.183]*	0.978 [0.599-1.594]	0.777 [0.189-3.189]	0.538 [0.169-1.708]	*0.627 [0.377-1.041]*	-*	1.352 [0.181-10.077]	-*
**Breastfeeding**								
No	1.0	1.0	1.0	1.0	1.0	1.0	1.0	-*
Yes	1.276 [0.847-1.922]	1.184 [0.765-1.834]	0.693 [0.169-2.844]	0.455 [0.144-1.443]	0.917 [0.597-1.409]	**3.561 [1.077-11.778]**	1.250 [0.168-9.321]	-*

*odds ratio could not be estimated.

Bold values are statistically significant ($p<0.05$); *italics* are borderline statistically significant (p value between 0.05 and 0.1)

Analysis encompassed multivariate binary logistic regression with goal type as the dependent variable (set goal type, did not set goal type). Sex, age, BMI, pregnancy, breastfeeding, and self-reported activity were independent variables. Analysis was conducted with SPSS version 23 (IBM corp, Armonk, NY). Analysis included n=7,979 users as it excluded n=88 users who had either an implausible BMI or an unspecified activity level.

	**Self-monitoring**	**Eat more of certain nutrients**	**Eat less of certain nutrients**	**Follow Canada's Food Guide Misc**	**Generic Healthy Eating**
**Sex**					
Female	1.0	1.0	1.0	1.0	1.0
Male	0.758 [0.408-1.409]	*0.495 [0.236-1.037]*	1.102 [0.503-2.411]	1.208 [0.848-1.722]	**0.696 [0.497-0.976]**
**Age**	1.008 [0.993-1.024]	1.006 [0.991-1.022]	**1.034 [1.012-1.057]**	0.997 [0.987-1.007]	**1.010 [1.002-1.018]**
**BMI**	**1.050 [1.026-1.075]**	**0.942 [0.905-0.981]**	0.990 [0.944-1.038]	0.993 [0.974-1.014]	0.988 [0.970-1.005]
**Activity**					
Sedentary	1.0	1.0	1.0	1.0	1.0
Low Active	0.790 [0.446-1.399]	*0.568 [0.299-1.077]*	0.721 [0.294-1.766]	1.043 [0.709-1.534]	1.175 [0.822-1.678]
Active	1.163 [0.608-2.226]	0.882 [0.456-1.710]	1.149 [0.440-3.001]	**0.625 [0.392-0.994]**	1.279 [0.865-1.891]
Very Active	1.908 [0.670-5.435]	1.599 [0.643-3.977]	2.534 [0.686-9.369]	0.814 [0.379-1.747]	**2.259 [1.304-3.913]**
**Pregnant**					
No	1.0	1.0	1.0	1.0	1.0
Yes	0.798 [0.108-5.876]	1.606 [0.385-6.699]	2.340 [0.312-17.542]	1.323 [0.480-3.647]	1.344 [0.582-3.100]
**Breastfeeding**					
No	1.0	1.0	-*	1.0	1.0
Yes	1.634 [0.390-6.837]	0.707 [0.096-5.185]	-*	0.513 [0.125-2.101]	0.766 [0.280-2.101]

*odds ratio could not be estimated.

Bold values are statistically significant ($p<0.05$); *italics* are borderline statistically significant (p value between 0.05 and 0.1)

Analysis encompassed multivariate binary logistic regression with goal type as the dependent variable (set goal type, did not set goal type). Sex, age, BMI, pregnancy, breastfeeding, and self-reported activity were independent variables. Analysis was conducted with SPSS version 23 (IBM corp, Armonk, NY). Analysis included n=7,979 users as it excluded n=88 users who had either an implausible BMI or an unspecified activity level.

	Weight loss	Weight gain	Weight maintenance/ management	Body composition	Disease management	Improve fitness	Be healthier
**Sex**							
Female	1.0	1.0	1.0	1.0	1.0	1.0	1.0
Male	**0.724 [0.608-0.862]**	**11.003 [5.924-20.439]**	**0.409 [0.219-0.761]**	**1.726 [1.228-2.426]**	1.251 [0.850-1.842]	1.211 [0.769-1.907]	*0.511 [0.245-1.070]*
**Age**	**1.014 [1.010-1.018]**	0.998 [0.975-1.023]	**1.031 [1.019-1.043]**	**0.981 [0.970-0.993]**	**1.064 [1.051-1.077]**	0.992 [0.979-1.006]	**1.020 [1.004-1.036]**
**BMI**	**1.025 [1.017-1.034]**	**0.656 [0.592-0.727]**	**0.969 [0.942-0.997]**	**0.935 [0.905-0.965]**	1.006 [0.985-1.028]	0.995 [0.965-1.026]	1.012 [0.982-1.044]
**Activity**							
Sedentary	1.0	1.0	1.0	1.0	1.0	1.0	1.0
Low Active	1.142 [0.959-1.359]	0.752 [0.281-2.016]	**2.445 [1.217-4.914]**	1.024 [0.541-1.940]	**0.639 [0.426-0.958]**	*2.269 [0.962-5.353]*	0.698 [0.374-1.303]
Active	*1.209 [0.991-1.475]*	0.752 [0.277-2.041]	**2.843 [1.365-5.921]**	**2.657 [1.433-4.927]**	0.770 [0.479-1.236]	**3.594 [1.495-8.636]**	1.199 [0.611-2.353]
Very Active	0.926 [0.630-1.360]	1.312 [0.402-4.281]	*2.685 [0.924-7.800]*	**4.020 [1.955-8.265]**	1.658 [0.781-3.518]	**9.133 [3.445-24.212]**	1.787 [0.615-5.197]
**Pregnant**							
No	1.0	-*	1.0	1.0	1.0	-*	-*
Yes	**0.233 [0.095-0.574]**	-*	0.555 [0.076-4.037]	0.377 [0.052-2.741]	2.488 [0.755-8.200]	-*	-*
**Breastfeeding**							
No	1.0	1.0	1.0	1.0	1.0	1.0	-*
Yes	1.333 [0.847-2.098]	3.052 [0.388-24.025]	0.517 [0.071-3.755]	1.450 [0.450-4.674]	1.395 [0.330-5.895]	0.624 [0.086-4.531]	-*

*odds ratio could not be estimated.
Bold values are statistically significant ($p<0.05$); *italics* are borderline statistically significant (p value between 0.05 and 0.1).

Analysis encompassed multivariate binary logistic regression with goal type as the dependent variable (set goal type, did not set goal type). Sex, age, BMI, pregnancy, breastfeeding, and self-reported activity were independent variables. Analysis was conducted with SPSS version 23 (IBM corp, Armonk, NY). Analysis included n=7,979 users as it excluded n=88 users who had either an implausible BMI or an unspecified activity level.
